# Choroid plexus carcinoma with leptomeningeal spread in an adult: a case report and  review of the literature

**DOI:** 10.1186/s13256-021-02887-2

**Published:** 2021-05-23

**Authors:** In Young Jo, Seung-Gu Yeo, Hyuk-Jin Oh, Jae-Sang Oh

**Affiliations:** 1 Department of Radiation Oncology, Soonchunhyang University College of Medicine, Cheonan, Republic of Korea; 2 Department of Neurosurgery, Soonchunhyang University College of Medicine, Cheonan, Republic of Korea

**Keywords:** Choroid plexus carcinoma, Leptomeningeal spread, Radiotherapy, Case report

## Abstract

**Background:**

Choroid plexus carcinoma is an intraventricular neoplasm originating from the choroid plexus epithelium and is of rare occurrence in adults. However, owing to the low prevalence of choroid plexus carcinoma, there is very limited information about the disease entity and treatment. Here we report a rare case of choroid plexus carcinoma in an adult patient.

**Case presentation:**

A 46-year-old South Korean (East Asian) male presented with low back pain, headache, and diplopia. Magnetic resonance imaging demonstrated enhancing mass lesion in the left trigone, cerebellar with leptomeningeal spread. Surgery was performed via left parietal craniotomy, and the lesion was histologically confirmed to be choroid plexus carcinoma. The patient received adjuvant craniospinal irradiation for remnant mass and leptomeningeal spread. Magnetic resonance imaging performed immediately after completion of the treatment revealed a partial decrease in the size of the tumor. However, the patient expired died as a result of acute respiratory distress syndrome before follow-up of long-term outcome.

**Conclusion:**

Choroid plexus carcinoma with leptomeningeal spread in adults is very important for rapid diagnosis and treatment. In the case of the presence of leptomeningeal spread, craniospinal irradiation can be considered as a treatment method, but may have serious complications. Hence, the technique should be applied with care.

## Background

Choroid plexus carcinoma (CPC) is rare intraventricular neoplasm originating from the choroid plexus epithelium. CPC is categorized as grade III according to the 2016 World Health Organization (WHO) classification, which is based on histological characteristics (cellularity, mitotic activity, pleomorphism, necrosis, and endothelial proliferation), and it is the most aggressive among choroid plexus tumors (CPTs) [1]. The incidence of CPTs is ~0.4–1% with probable occurrence within less than 2 years of life [2]. Most of the CPCs reported so far are in pediatric patients and have been rarely reported in adult patients [3–8]. Here, we report a case of CPC with leptomeningeal spread in a 46-year-old man.

## Case presentation

**Fig. 1:**
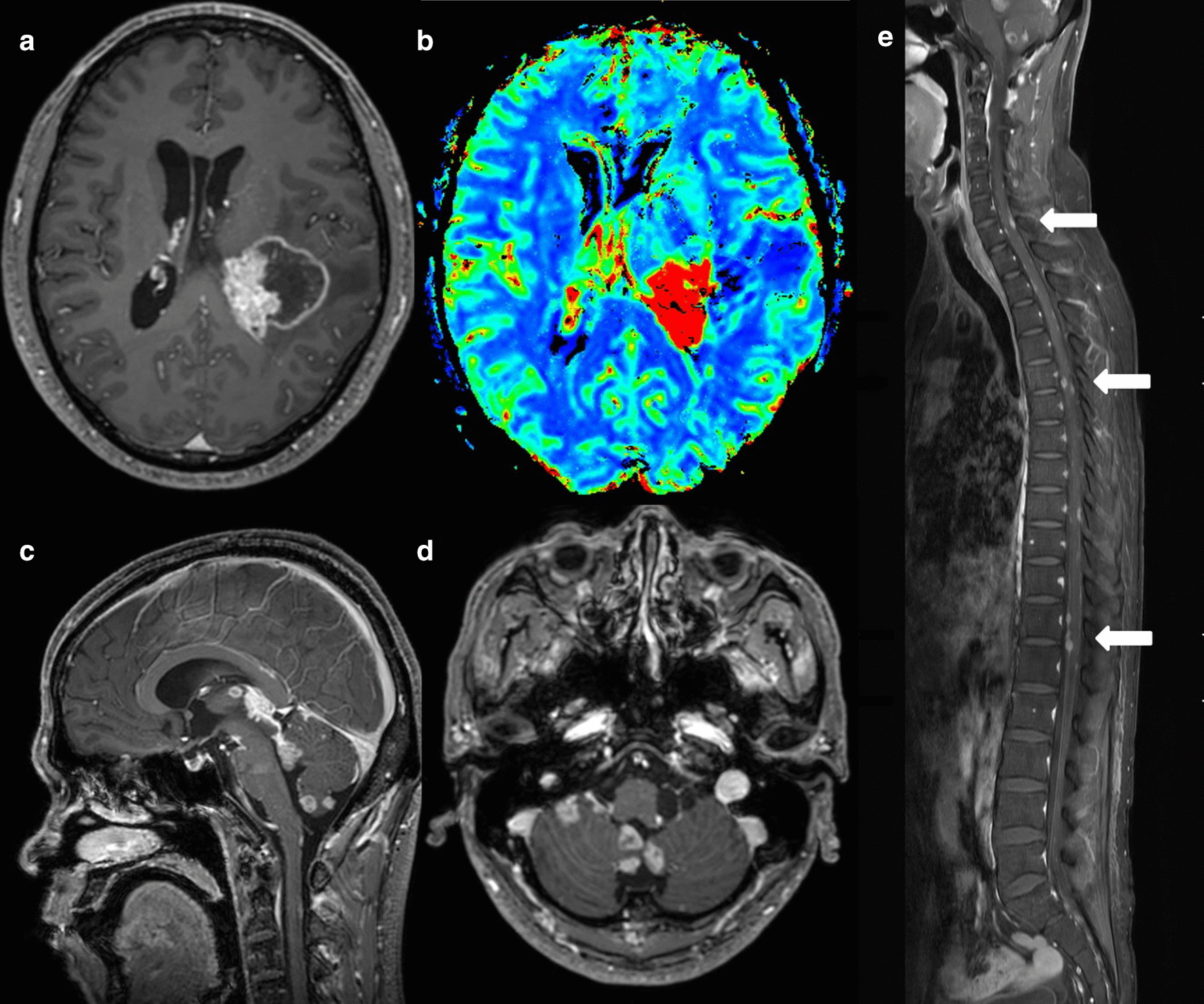
Preoperative magnetic resonance imaging scans (**a**–**e**). Brain MRI with gadolinium enhancement revealing heterogeneous enhancing masses in the left thalamus, left parietotemporal lobe, posterior part of left lateral ventricle, left temporal horn, interpeduncular cistern, quadrigeminal cistern, and right cerebellopontine angle cistern. **a**, **c**, **d** Perfusion MRI showing increase in cerebral blood flow at the mass lesion. **b** Spine MRI revealing multiple linear and nodular contrast-enhancing lesions (white arrows) within the cord and leptomeninges in the entire spine area (**e**)

**Fig. 2:**
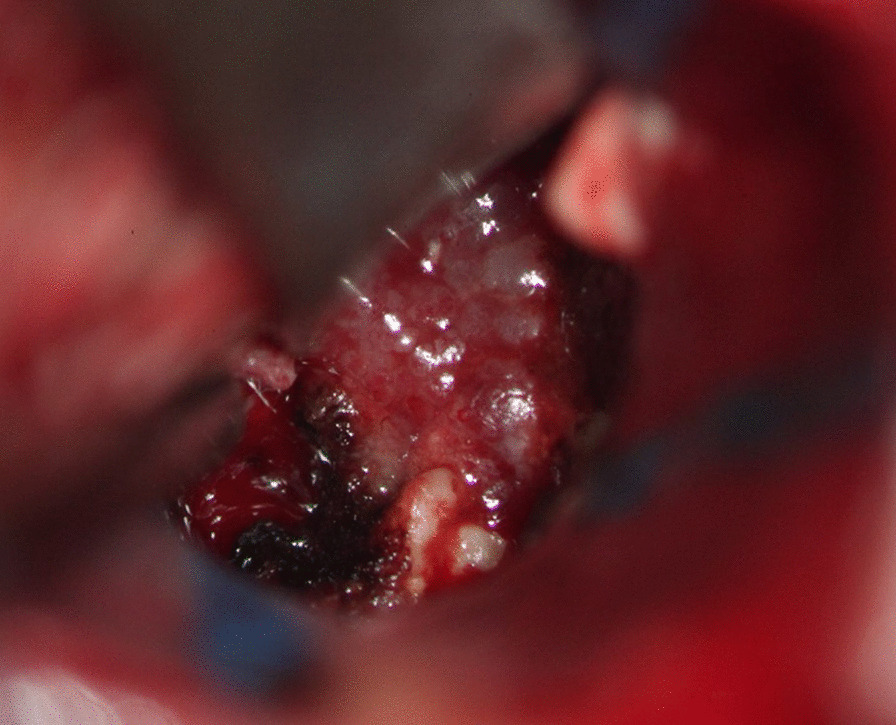
Intraoperative view. The tumor was a mildly firm, pinkish mass with calcifications

**Fig. 3:**
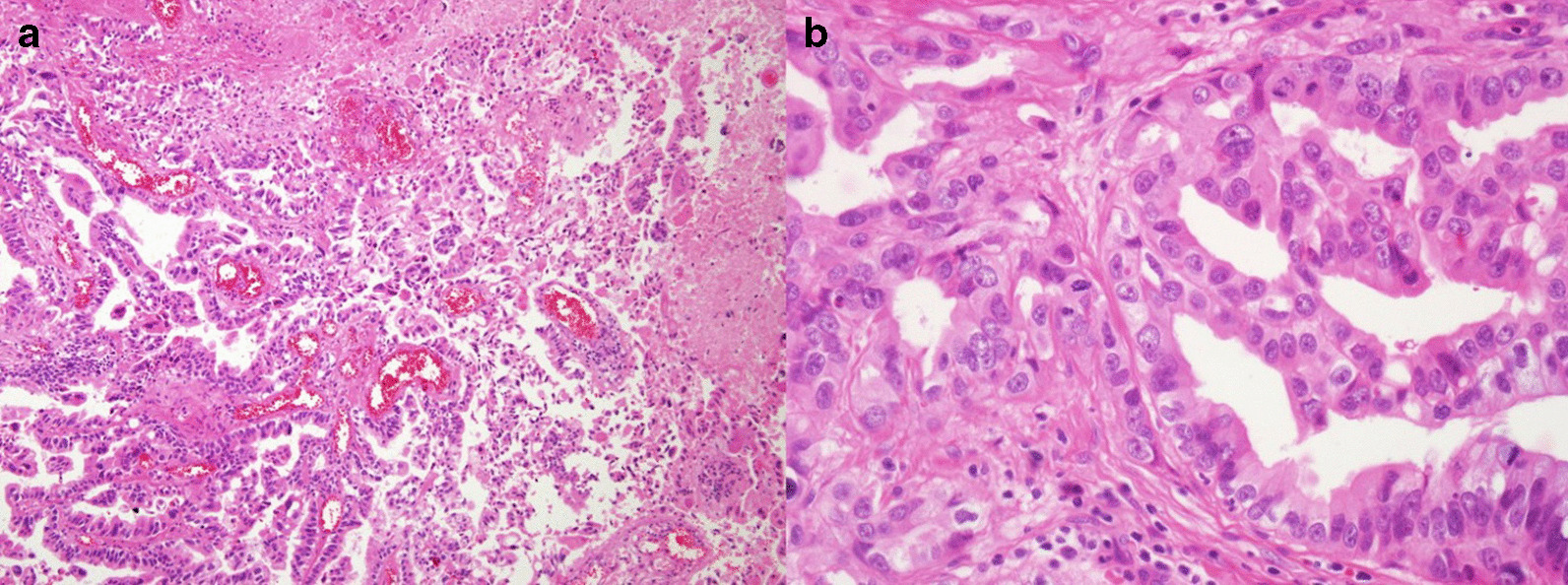
Histologic features of choroid plexus carcinoma. **a** Tumor characterized by papillary growth and extensive necrosis. **b** High-power view showing atypical tumor cells with increased mitosis

This case report was approved by the Institutional Review Board of Soonchunhyang University Cheonan Hospital (approval no. SCHCA 2018-05-051), which waived the requirement for informed consent. A 46-year-old South Korean (East Asian) man visited the hospital with back pain that started 3 months back. The patient had several supportive therapies for sustained low back pain at another hospital, but the treatments were not effective. When the patient first visited the hospital, he complained of headache, diplopia, and sensory change of right arm and face in addition to low back pain. Multiple nodular mass lesions were observed around cauda equina on spine magnetic resonance imaging (MRI) taken at the other hospital. MRI with gadolinium enhancement revealed heterogeneous enhancing masses in the left cerebrum, bilateral cerebellum, and whole spinal canal, in particular diffuse wall enhancement in posterior and temporal horn of left lateral ventricle. Diffusion and perfusion MRI showed increased cerebral blood flow and high cellularity in the left cerebral mass lesions (Fig 1). ^18^F-fluorodeoxyglucose positron emission tomography scan showed no hypermetabolic lesions, thereby ruling out suspicious primary neoplasms. The patient underwent surgery via left occipital craniotomy. The tumor was a mildly firm, pinkish mass with calcified multilobulations (Fig. 2). The medial part of the mass was calcified and well-bounded, but the other part invaded normal brain parenchyma. A subtotal resection of the solid tumor was achieved, and histologic examination was performed. Papillary architecture lined by atypical tumor cells and extensive necrosis was noted (Fig. 3a). A high-power view demonstrated highly atypical tumor cells with increased mitosis (Fig. 3b). We performed additional immunostaining, showing that CK7 was positive, CK20 was negative, and P53 was strongly positive. TTF-1 immunodeficiency dyeing was performed to distinguish metastatic central nervous system (CNS) adenocarcinoma of the lung. Consequently, the probability of metastatic adenocarcinoma originating in the lung was ruled out, because TTF-1 was negative. Based on these observations, the patient was diagnosed with CPC, WHO grade III.

**Fig. 4:**
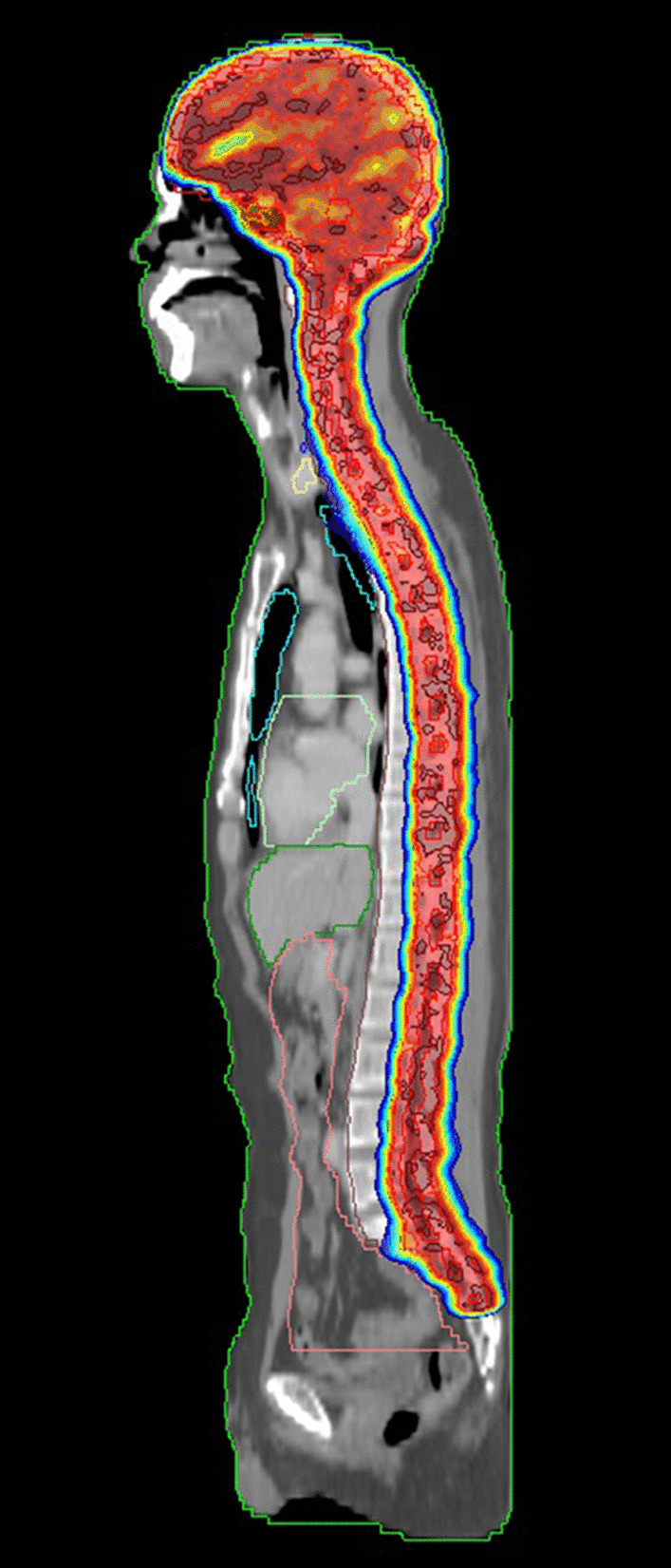
Craniospinal irradiation with intensity-modulated radiation therapy (IMRT) plan. The brain and spinal canal were irradiated with normal organ sparing

Postoperative adjuvant radiation therapy was performed. The patient underwent a simulation computed tomography (CT) scan with contrast enhancement at the supine position. Craniospinal irradiation (CSI) treatment was planned divided into three parts: brain, upper spine, and lower spine. To minimize bone marrow suppression, we proceeded to the intensity-modulated radiation therapy (IMRT) plan (Fig. 4). After ten fractions of CSI, gap moving was performed to prevent a hot-spot region from occurring in the field gap. CSI was performed up to a total of 36 Gy in 20 fractions followed by boost radiation therapy of 18 Gy in 10 fractions for a remnant lesion in the brain. The patient showed improvement of motor grade of lower extremities from grade 1-2 to 2-3 at the end of radiotherapy. After the end of treatment, follow-up MRI images taken during radiation therapy revealed a mild decrease in tumor size. The patient completed the CSI dose scheme without any special side effects except for a period of 2 days during radiation therapy because of intermediated bone marrow suppression. During this period, absolute neutrophil count (ANC) decreased to 840/μl, and granulocyte-colony stimulating factor(G-CSF) was injected. After completion of the treatment, the patient underwent rehabilitation. While considering adjuvant chemotherapy, aspiration pneumonia occurred. Unfortunately, the patient died 4 months after diagnosis and surgery, or 1 month after radiotherapy completion, as a result of acute respiratory distress syndrome (ARDS).

## Discussion and conclusion

Choroid plexus carcinoma is a very rare cancer with a poor prognosis that occurs most commonly in children, with and the incidence in adults being much less [9]. The patient reported in this paper was in his mid-40s with no previous medical history. It is well known that maximal surgical resection in CPC majorly influences the overall survival and progression-free survival [2, 10, 11]. However, CPC is often accompanied by leptomeningeal seeding at the time of initial diagnosis because of its ability to spread well into the spinal canal along the ventricle. In this report, the tumor masses of the patient were disseminated in the brain and spinal cord canal at the time of the initial diagnosis. Adjuvant radiation therapy was performed about 3 weeks after surgery.

A few studies have reported that radiation therapy leads to better clinical outcome in patients with CPC [2, 11–13]. However, the radiation field of CPC is a topic of debate. In this patient, CSI followed by boost RT was processed because the gross tumor had spread to the whole spinal canal, and tumor progression outside the treatment field was more than half. Mazloom *et al*. reported that CSI has better overall survival and progression-free survival rate than whole-brain radiation therapy (WBRT) or involved-field radiation therapy (IFRT), with statistical significance [10]. Furthermore, Fabi *et al*. concluded that CSI could be more effective than chemotherapy when leptomeningeal seeding is present. Even in our case, radiation therapy was found to be effective for CPC, but it is contemplated that further consideration of the optimal radiation dose and treatment field will be necessary [4].

Although the best chemotherapy regimen for CPC is still unknown, a report revealed long-term outcomes while applying temozolomide (TMZ) in CPC patient with MGMT methylation, while another report showed no effect of TMZ in the absence of MGMT methylation [4, 5, 14]. In our case, TMZ-based chemotherapy did not proceed in the patient because MGMT methylation was negative in the patient. In the near future, a comparative study of TMZ with etoposide, which has been proven effective in a previous study, is also needed [15]. Also, it is necessary to study the role of intrathecal chemotherapy [2]. As it is difficult to perform randomized controlled trials on rare carcinomas such as CPC, diverse experiences of CPC in different institutions should be reported and shared. In particular, additional research on optimal radiation field and dose and suitable chemotherapy regimen is needed.

## Data Availability

The datasets used and/or analyzed during the present study are available from the corresponding author on reasonable request.

## Abbreviations

CPC: Choroid plexus carcinoma
LMS: Leptomeningeal spread
CSI: Craniospinal irradiation
ARDS: Acute respiratory distress syndrome
CPTs: Choroid plexus tumors
IMRT: Intensity-modulated radiation therapy
TMZ: Temozolomide
